# COVID-19 infection as a trigger for new-onset type 1 diabetes in a susceptible individual – or just coincidence?

**DOI:** 10.3205/dgkh000388

**Published:** 2021-04-22

**Authors:** Robert Kästner, Igor Alexander Harsch

**Affiliations:** 1 Department of Pediatrics, Thuringia Clinic “Georgius Agricola”, Saalfeld/Saale, Germany; 2 Department of Internal Medicine II, Thuringia Clinic Saalfeld “Georgius Agricola”, Saalfeld/Saale, Germany

**Keywords:** COVID-19, type 1 diabetes mellitus, celiac disease, attention deficit hyperactivity disorder

## Abstract

**Background:** The cytotoxic effects of COVID-19 raise the question of a possible relation between COVID-19 infection and new-onset type 1 diabetes. We report the case of an eight-year-old boy with new-onset type 1 diabetes and an asymptomatic COVID-19 infection.

**Case presentation:** The eight-year-old boy was hospitalized on December 18^th^, 2020 due to increased polyuria during the preceding 1 week. Type 1 diabetes was diagnosed with autoantibodies against glutamic acid decarboxylase, tyrosine phosphatase and insulin. The Hba1c value was 11.6%. Upon admission, the PCR test for COVID-19 was positive, the duration of the infection was not clear due to the asymptomatic course, and antibodies were initially negative. Significantly elevated antibodies against COVID-19 were detected 15 days later.

**Conclusion:** The laboratory findings led us to the hypothesis that the boy already had an increased risk of developing autoimmune diseases (HLA DR3-DQB1*02:01 and DR4-DQB1*03:02 positive). The Hba1c value allows speculation that the diabetes manifestation was already “on the way” and that a relatively recent COVID-19 infection could have accelerated the process. The findings are in contrast to a recent report in which COVID-19 infection preceded the manifestation of an insulin-dependent diabetes mellitus by about 5–7 weeks. Due to the paucity of reports, cases with a suspected connection between diabetes mellitus and COVID-19 infection should be entered into the CoviDiab registry (https://covidiab.e-dendrite.com).

## Zusammenfassung

**Hintergrund:** Die zytotoxischen Wirkungen von COVID-19 werfen die Frage nach einem möglichen Zusammenhang zwischen der COVID-19-Infektion und neu auftretendem Typ-1-Diabetes auf. Wir berichten über den Fall eines achtjährigen Jungen mit neu aufgetretenem Typ-1-Diabetes und asymptomatischer COVID-19-Infektion.

**Falldarstellung:** Der achtjährige Junge wurde am 18. Dezember 2020 wegen zunehmender Polyurie seit 1 Woche ins Krankenhaus eingeliefert. Der Typ-1-Diabetes wurde klinisch und mit Autoantikörpern gegen Glutaminsäure-Decarboxylase, gegen Tyrosinphosphatase und Insulin diagnostiziert. Der Hba1c-Wert betrug 11,6%. Bei Aufnahme war die PCR für COVID-19 positiv, die Dauer der Infektion war aufgrund des asymptomatischen Verlaufs nicht klar. Die Antikörper gegen COVID-19 waren initial negativ. 15 Tage später wurden signifikant erhöhte Antikörper gegen COVID-19 nachgewiesen.

**Schlussfolgerung:** Die Laborergebnisse führten uns zu der Hypothese, dass der Junge bereits ein erhöhtes Risiko für die Entwicklung von Autoimmunerkrankungen hatte (HLA DR3-DQB1*02:01 und DR4-DQB1*03:02 jeweils positiv). Der Hba1c-Wert lässt Spekulationen zu, dass die Diabetes-Manifestation bereits „auf dem Weg“ war und dass eine relativ kürzliche COVID-19-Infektion den gesamten Prozess beschleunigt hat. Die Ergebnisse stehen im Gegensatz zu einem 2020 veröffentlichten Bericht, in dem eine COVID-19-Infektion etwa 5–7 Wochen vor der Manifestation eines insulinpflichtigen Diabetes mellitus auftrat. Aufgrund des Mangels an Berichten sollten Fälle mit Verdacht auf einen Zusammenhang zwischen Diabetes mellitus und COVID-19-Infektion in das CoviDiab-Register (https://covidiab.e-dendrite.com) eingepflegt werden.

## Introduction

During the first two months of the COVID-19 pandemic in Germany, Kamrath et al. [1] reported a significant increase in diabetic ketoacidosis and severe ketoacidosis upon the diagnosis of type 1 diabetes in children and adolescents. The authors discussed underlying causes as potentially multifactorial and as a reflection of reduced medical services, fear of approaching the health care system, and more complex psychosocial factors. Another possible reason for an increase of such cases was recently highlighted by Hollstein et al. [2]. The authors reported the case of a 19-year-old male with newly diagnosed type 1 diabetes, who had an asymptomatic SARS-CoV-2 infection 5–7 weeks previously. Although one case is certainly not a proof of causality, several factors (no serum antibodies against islet cells, glutamic acid decarboxylase, insulin, zinc-transporter 8 and tyrosine phosphatase, as well as no human leucocyte antigen genotype highly susceptible for the development of diabetes mellitus) make it worthy of discussion. This is why we now report the case of an eight-year-old boy with new-onset type 1 diabetes and COVID-19 infection.

## Case report

An eight-year-old boy was hospitalized on December 18^th^, 2020 because of increasing polyuria during the 1 week prior to admission. The child had lost 1 kg. Ketone bodies (>15 mmol/l) at a pH of 5 and glucosuria of 56 mmol/l were detected in the urine. The glucose value in capillary blood was 20.5 mmol/l (=369 mg/dl). The Hba1c level was 11.6%, corresponding to 103 mmol/mol H (29–42). Since testing for COVID-19 is currently mandatory for new admissions in our clinic, the rapid antigen test was found positive and was then confirmed by a positive PCR. There were no symptoms of an infection of the upper respiratory tract.

Starting on December 17^th^, 2020, the child’s mother noticed a loss of olfaction, which was later accompanied by flu-like symptoms. Her PCR for COVID-19 was also positive.

Interestingly, the child already had a possible history of celiac disease that was diagnosed in a children’s hospital at the age of 2.5 years due to meteorism and diarrhea. (According to the old hospital record, Gliadin IgG antibodies [EIA] were 61.19 RE/ml [<25] and Gliadin IgA antibodies were 3.18 RE/ml [<25]. Unfortunately, transglutaminase IgG and IgA antibodies had not been measured at the time and controls had not been done.) Due to dietary measures, the child was free of symptoms, and surprisingly, the diet could be discontinued at an age of 7 years. Attention deficit hyperactivity disorder (ADHD) was diagnosed a few months ago. There is a family history of diabetes mellitus insofar as a great-aunt had died of diabetes at the age of 18.

## Laboratory findings

- Autoantibodies relevant for diabetes mellitus type 1:
  - against glutamic acid decarboxylase (GAD): 1172 IU/ml (<10),
  - against tyrosine phosphatase (IA-2-Ab): >4,000 IU/ml (<19),
  - insulin-antibodies 4.81 U/ml (<0.4),
  - against islet cells (IFT): negative,
  - against zinc-transporter 8 (ZnT8-Ab): negative.
- C-peptide: 0.80 µg/l (0.49–5.52).
- Human leucocyte antigens:
  - HLA DR3-DQB1*02:01: positive;
  - DR4-DQB1*03:02: positive;
  - DQB1*0:6:02: negative.
  - Assessment: The patient is a carrier of predisposing alleles and has a significantly higher risk of developing type 1 diabetes compared to persons with negative characteristics. The protective allele is negative.
- Antibodies relevant for celiac disease: the serologic tests for the three antibodies common in celiac disease: anti-tissue transglutaminase (tTG) antibodies, endomysial antibodies (EMA) and deamidated gliadin peptide (DGP) antibodies were negative. IgA: 1.36 g/dl (0.34–3.05). Furthermore, autoantibodies against thyroid antigens were not detected.
- Antibody testing against COVID-19 (IgG and IgA) was negative on December 22^nd^, 2020. On January 3^rd^, 2021, the PCR for COVID 19 was negative. On January 5^th^, IgA was 7.11 (<0.8) and IgG was 3.29 (<0.8).

Course: During the first day, the patient was administered intravenous insulin therapy, on day 2 subcutaneous insulin therapy with an intensified conventional regimen (total insulin requirement: 28 IU) was initiated. Over the course of the next few days, blood glucose levels stabilized at 8.4–10.3 mmol/l. The patient and his parents received an educational program (~70% telemedical) according to the guidelines for insulin-dependent diabetes mellitus management. The patient further received a continuous glucose measurement system (CGMS; FreeStyle Libre 2 System, Abbott Diabetes Care). He was discharged in a good state, and the total insulin requirement was about 15 IU.

## Discussion

Viruses have long been discussed as candidate triggers of islet autoimmunity and type 1 diabetes based on epidemiological and experimental evidence. Enterovirus infection is frequently discussed, but some studies have also reported potential associations of other viruses with type 1 diabetes, including mumps, cytomegalovirus, rotavirus, parechovirus, Epstein-Barr virus, rubella, and parvovirus [3]. The COVID-19 virus has been recently discussed as a further candidate [2].

In the first wave of the COVID-19 pandemic, patients were not regularly tested for COVID-19. Thus, some possible coincidences between diabetes manifestation and the infection were not detected in patients who were asymptomatic with regard to COVID-19.

Unfortunately, due to the boy's asymptomatic course, it is not possible from biographical data to clarify how long he already had the COVID-19 infection.

Looking at the laboratory parameters as a whole, it is possible to initially speculate the following: The boy already had an increased risk of developing autoimmune diseases (see biographical information on celiac disease and the information that a great-aunt had died of diabetes at the age of 18, as well as the HLA status). The Hba1c value allows speculation that the event was already “on the way” and that a relatively recent COVID-19 infection (see antibody history) could have accelerated the whole process.

As for the increased risk for developing autoimmune disease, the association between celiac disease and type 1 diabetes is well known [4]. Unfortunately, the diagnosis of celiac disease in our case was not clearly established according to the recommended ACG guidelines [5]. However, according to the mother’s report, the condition seems very likely; attempts since the putative diagnosis to re-institute a normal diet led to meteorism and diarrhea. The finding of a “remission” at the age of 7 does not necessarily exclude the diagnosis. In a retrospective analysis by Matysiak-Budnik et al. [6], the authors reported that, in a long-term follow-up of 61 patients with celiac disease diagnosed in childhood, evolution toward latency is possible on a normal diet in some cases.

Nonetheless, the case report has limitations. The causality between COVID-19 infection and the onset of type 1 diabetes cannot be proven. 

We know nothing about the speed at which COVID-19 can destroy human beta cells, especially not in a potentially susceptible individual. Thus, next to the possibility that nascent type 1 diabetes mellitus with an acceleration of the process through the cytotoxic effects of COVID-19 occurred, the infection could also have been the primary trigger. The elevated Hba1c does not necessarily mean that hyperglycemia really preceeded COVID-19 infection: variations in red blood cell survival are known that may cause a variation in Hba1c levels for a given mean blood glucose [7], as the authors of the case of the 19-year-old male with newly diagnosed type 1 diabetes also claim [2].

It is in the nature of casuistic reports not to be able to clarify causality. In addition, they cannot rule out simple coincidence, since COVID-19 infection is frequent in the population. For this reason, the CoviDiab registry was recently put online, in which cases with a suspected connection between diabetes mellitus and COVID-19 infection should be entered: https://covidiab.e-dendrite.com. 

## Conclusions

Due to the cytotoxic potential of the COVID-19 virus, negative effects on the pancreatic beta-cells seem possible. In contrast to a recent report, the case described here suggests an acceleration of the problem in an already predisposed individual. To date, there are very few reports; collection and reporting in a registry is mandatory to gain more information about a possible association between COVID-19 infection and insulin-dependent diabetes mellitus.

## Notes

### Competing interests

The authors declare that they have no competing interests.

### Funding

There was no financial support.

### Patient’s consent

The patient’s parents gave informed consent to the reporting of their child’s case.

### Acknowledgement

The authors would like to thank Kerstin Kaiser (diabetes advisor DDG) for the care and training of the child and his parents as well as the grandparents under the extraordinary circumstances described.
